# Establishing the relationship between nurse practitioner integration and reductions in health disparities

**DOI:** 10.1186/s12912-025-04190-7

**Published:** 2025-12-04

**Authors:** Abigail Kra-Friedman, Rebecca Clark, Branden Dutchess, Nancy P. Blumenthal, Moriah E. Ellen, Joshua Porat-Dahlerbruch

**Affiliations:** 1 Department of Nursing, Faculty of Life and Health Sciences, Jerusalem College of Technology, Lev Academic Center, 21 Ha-Va’ad Ha-Leumi St, Jerusalem, Israel; 2 School of Nursing, University of Pennsylvania, 418 Curie Blvd, Philadelphia, PA 19104 USA; 3 Center for Health Outcomes & Policy Research, 418 Curie Blvd, Philadelphia, PA 19104 USA; 4 Pennsylvania Hospital, 800 Spruce Street, Philadelphia, PA 19107 USA; 5 Department of Acute & Tertiary Care, School of Nursing, University of Pittsburgh, 336 Victoria Building, 3500 Victoria Street, Pittsburgh, PA 15261 USA; 6 Department of Health Policy and Management, Faculty of Health Sciences and Guilford Glazer Faculty of Management, Ben-Gurion University of the Negev, P.O. Box 653, Be’er Sheva, 841051 Israel; 7 Israel Implementation Science and Policy Engagement Centre, Ben-Gurion University of the Negev, P.O. Box 653, Be’er Sheva, 841051 Israel; 8 Institute of Health Policy Management and Evaluation, Dalla Lana School of Public Health, University of Toronto, 155 College St, Room 500, Toronto, ON Canada

**Keywords:** Advanced practice nursing, Health equity, Health workforce, Healthcare disparities, Implementation science, Nurse practitioners, Nursing, Workforce

## Abstract

**Background:**

Nurse practitioners are widely recognized for their holistic, patient-centered approach, which addresses the needs of patients, their families, and the communities they serve. Nurse practitioner care is noted internationally for reducing health disparities by improving care access and quality for underserved populations. However, there is limited empirical evidence on how well nurse practitioners are integrated into care models, which impacts the nurse practitioner workforce’s ability to reduce health disparities. This study aimed to explore how the extent of the integration of nurse practitioners within care models influences health disparities.

**Methods:**

This was part of a larger Delphi study guided by the Nurse Practitioner Integration Model. Open-ended surveys were sent to scholars and practice experts with experience in nurse practitioner integration. Data were collected from 29 participants. Inductive content analysis and member checking ensured the rigor and credibility of the results.

**Results:**

Two core themes emerged: First, *poor integration and limited role autonomy in the collaborating organization restrict nurse practitioner capacity to promote equity and decrease disparities*, largely due to organizational policies that fail to prioritize nurse practitioner integration. Second, *the distinct nurse practitioner approach is an untapped opportunity to increase equity and reduce disparities in low-integration settings*, whereby well-integrated nurse practitioners could significantly impact outcomes in underserved populations.

**Conclusions:**

Effective nurse practitioner integration is crucial for advancing the United Nations Sustainable Development Goals, which emphasize improving health and wellbeing for all through diminished disparities. Policies and investment should prioritize full integration of nurse practitioners into care teams and organizations, maximizing their holistic, patient-centered approach to care and leveraging their unique contributions to expand access and promote health equity for underserved populations.

## Background

Nurse practitioners (NPs) are health care providers educated to apply a holistic approach to care, accounting for patient, family, social, and community factors [1, 2]. Studies from multiple countries demonstrate that NP care is associated with improved quality of and access to care for populations affected by intersectional health inequalities, such as racial/ethnic, sex, sexual identity, age, disability, socioeconomic status, and geographical disparities [2–7]. In accordance with the international literature, the World Health Organization’s 2025 State of the World’s Nursing [8] report highlights the vital contribution of NPs for improving access to healthcare, specifically in vulnerable populations and underserved geographic areas. The report, furthermore, suggests that a lack of NP services may be a missed opportunity to address many of the UN Sustainable Development Goals, including good health and wellbeing (Goal 3) and reducing inequalities (Goal 10). However, implementing NPs in a care setting does not guarantee reductions in health disparities [9, 10]. Meaningful impact depends on effective integration [9]. NP integration is defined as “a multi-level process of incorporating NPs into a care model to the extent that they can function to their full scope of practice and education, which leads to improved patient, health system, and population needs” [10, 11]. Successful NP integration is dependent on several factors, including the regulatory scope of practice, resource availability, and interprofessional collaboration [12–14]. Without thoughtful and intentional integration policies, the full potential of NPs to advance equity and health outcomes may not be realized fully [9]. Instead of focusing on the impact of NP integration on reductions in health disparities, most of the literature focuses on the association between the presence of NPs and reduced health disparities [15, 16]. Large-scale analyses examining thousands of primary care practices across diverse U.S. communities have shown that NPs are disproportionately present in areas marked by socioeconomic disadvantage, demonstrating a broad pattern in which NPs help fill critical gaps in access where physician availability is lowest [17]. Similarly, extensive workforce research using multi-state Medicaid data has demonstrated that greater NP supply is associated with significant reductions in system-level spending on preventable emergency department visits and avoidable hospitalizations [18]. The scope and consistency of these findings across large, heterogeneous populations underscore how expanding NP workforce capacity can meaningfully reduce geographic, economic, and utilization-related disparities, strengthening health equity at the system level. These large-scale correlative studies, however, do not provide information on how NP care may reduce disparities nor how the NP integration process affects this link between NP presence and reduced disparities. Such information can provide practical guidance for managers and decision-makers aiming to reduce disparities in care outcomes by integrating NPs.

Three international literature reviews on NP integration have found that many healthcare systems intentionally choose to introduce NPs in efforts to provide improved care for underserved populations [11, 19, 20]. These reviews, moreover, discussed the slim chances of reducing inequalities without successful NP integration. Experts published a viewpoint reflecting on these three seminal reviews and suggested that the link between successful NP integration and reducing inequalities is not grounded in empirical evidence and instead has been assumed by researchers [9]. Finally, little research has focused on the mediating process of NP integration and reducing health disparities [21]. Taken together, the existing literature indicates a need for more evidence grounded in insights on how NP integration affects health inequalities.

Understanding the significance of the integration process is crucial for policymakers and organizational managers who aim to achieve improved health equity and reduced health disparities through NP care. This study aimed to explore how the integration of NPs within care models influences health disparities. Exploring this phenomenon may highlight the importance of developing policies that facilitate the effective integration of NPs into care models to reduce health inequities.

## Methods

### Study design

This qualitative descriptive study was part of a larger Delphi study aimed at developing an inventory of factors affecting NP integration [14]. The Delphi process was based on the guide by Belton et al. [22]. Conceptualization of this study was based on the NP Integration Model [10, 11] which theorizes that NP integration mediates the process between introducing NPs and achieving diminished health disparities. Further details on the study design can be found in the published parent study [14].

### Participant recruitment and setting

Participants were identified based on their level of knowledge about NP integration in the US. Both “traditional” and “non-traditional” experts were included. “Traditional” experts refer to NP practice experts and scholars with at least three publications on NP integration [23]. “Non-traditional” experts were included if they were either in the first group of NPs integrating into a care model or the primary individual responsible for integrating NPs within their organizational structure. An advisory panel of five individuals representing a subset of potential participants nominated study participants [23]. Panelists were asked to emphasize nominees with expertise in health disparity outcomes. The lead investigators reviewed nominees to ensure adherence to the inclusion criteria.

The potential participants received an email explaining the study’s aims, expected time commitment, ethical considerations, and a timeline for the study. Two follow-up emails were sent at two-week intervals if no response was received. After agreeing to participate, researchers verified that the participant self-identified as an expert in NP integration and disparities. Participant demographic information was collected at this stage via an online survey to ensure that a diverse set of participants agreed to participate [24].

### Data collection

Participants received a survey asking them to provide a written open response to the following prompt: “Based on your knowledge and/or experience, please describe how NP integration policies or the extent of NP integration affect the capacity for NPs to address health disparities.” Participants were provided with a definition of NP integration [11], as well as the definition of *health disparities* from Healthy People 2030 [25], which read: “Health disparity is a particular type of health difference that is closely linked with social, economic, and/or environmental disadvantage. Health disparities adversely affect groups of people who have systematically experienced greater obstacles to health based on their racial or ethnic group; religion; socioeconomic status; gender; age; mental health; cognitive, sensory, or physical disability; sexual orientation or gender identity; geographic location; or other characteristics historically linked to discrimination or exclusion.” Data were collected from September through October 2023. Participants were given six weeks to respond. Two follow-up reminders were sent if a participant had not yet responded. Following data analysis, participants were sent results for consensus voting (see Data Analysis section).

### Data analysis

The Guide for Qualitative Analysis in Health Services Research guided the design of the analysis [26]. Inductive content analysis was used [27]. Content analysis is an effective method for describing a conceptual issue through written feedback.

Two coders independently conducted a line-by-line review of all open-ended responses in qualitative software. The two coders met to develop an initial code structure (28) which was then applied to the data independently. Researchers used an iterative process to update the coding structure as analysis progressed. The two coders met weekly for peer debriefing to acknowledge and recognize any personal biases and traits that could impact data analysis interpretation [28]. Any unresolvable discrepancies were discussed with a third researcher [29]. The two researchers then synthesized codes into themes and descriptions. During theme induction, codes were analyzed in the context of the participants’ role: scholar or practice expert.

An audit trail was maintained to trace the induction from the data to themes. The advisory panel was shown the audit trail and provided suggestions as a measure to enhance trustworthiness [30]. Changes were made to the themes and descriptions accordingly.

The themes were sent to all participants for member checking [31]. The participants rated their level of agreement with the themes and descriptions. Open-ended feedback was requested. The two coders reviewed responses together, and changes were made based on feedback. An audit trail was maintained throughout. A third researcher independently reviewed the audit trail and revisions. Once those changes were made, the audit trail was reviewed by the advisory panel, and changes were made until a unanimous consensus was reached.

### Trustworthiness

We used the guidelines by Guba [32] to ensure rigor, which has been reiterated more recently by Ahmed [33]. Credibility and reliability were improved through member checking [31]. Reliability was addressed by switching the order of questions when sent back to participants for member checking [22, 34]. We also used content validity by asking an expert advisory panel to review the wording of our survey to ensure that we were asking what we intended to measure [22]. Finally, transferability was addressed by reporting the participant characteristics in the results.

### Ethical considerations

This study adhered to the ethical guidelines and standards outlined in the Declaration of Helsinki. Accordingly, this study was approved by the University of Pittsburgh Institutional Review Board (STUDY23040081). All participants provided consent to participate.

## Results

### Participant characteristics

Data from 29 participants were analyzed in this study out of 56 who were invited to participate (52% participation rate). Participant demographics are presented in Table 1. Of all participants, 41% were practice experts, and 59% were scholars. During the member checking phase, 25 of the 29 participants responded (86% response rate).

**Table 1:** Participant characteristics

Characteristic	Participants (No., %)
**Total**	29 (100%)
**Years of Experience**	
0–10	17 (59%)
11 to 20	5 (17%)
21+	7 (20%)
No response	1 (3%)
**Age**	
20–39	6 (20%)
41–60	12 (41%)
61+	7 (24%)
No response	4 (14%)
**Gender Identity**	
Male	4 (14%)
Female	25 (86%)
**Participant Type**	
Scholar/Researcher	17 (59%)
Practice Expert	12 (41%)
**Work Setting**	
University/Research Institute	21 (72%)
Healthcare Setting	8 (28%)

Two themes arose from the data: (1) *Poor integration and limited role autonomy in the collaborating organization restrict NP capacity to promote equity and decrease disparities*, and (2) *The distinct NP approach is an untapped opportunity to increase equity and reduce disparities in low-integration settings*. Below, we describe the themes, report the average level of participant agreement with themes and descriptions, and present exemplar quotes.

### Theme 1: Poor integration and limited role autonomy in the collaborating organization restrict NP capacity to promote equity and decrease disparities

This theme reflects participants’ views that care organizations often fail to integrate NPs efficaciously. A lack of full autonomy, patient panel ownership, and continuity of care can significantly affect NPs’ ability to address health disparities. Organizational protocols may impose restricted clinical privileges, assign responsibility solely for walk-in patients, enforce limitations based on physician preferences, and restrict acceptance of specific insurance plans.

Role design affects whether NPs practice to their full scope of practice. If NPs are used by organizations to fill gaps in service delivery rather than lead care, they are filling the needs of the organization, not the needs of the people. **Participant 8** noted: “*The capacity for NPs to address health disparities was diminished in one setting I practiced in which the role of the NP was that of ‘medical resident replacement’. NPs were utilized only to fill gaps in coverage… not to practice to the full extent of their unique expertise*.” **Participant 7** said: “*When a health system does not recognize or prioritize this unique disposition in their care delivery context*,* NPs aren’t always able to make the greatest use of their knowledge and resources - they’re too busy trying to get through their overbooked schedule and keep up on documentation requirements.*” Finally, **Participant 5** noted that, “*restrictions on scope of practice limit NPs from practicing in all [geographic] areas that would benefit from additional health care services*.”

Predetermined collaborative agreements with physicians may pose a barrier to NPs’ ability to provide care in underserved populations. As **Participant 10** noted: “*NPs are required*,* either by state or by the organization*,* to practice in a predetermined collaborative arrangement… [which] may hinder any focus on health disparities*.” **Participant 12** noted that system priorities (e.g., RVUs) may limit holistic care. She said, “*NPs who were pressured by their collaborating physicians and administrators to provide care at the maximum RVU possible*,* which dictated the manner in which NP care was provided.*” Finally, **Participant 6** stated that, “*if physicians choose not to accept certain patient insurances (e.g.*,* Medicare*,* Medicaid)*,* the collaborating NP cannot accept patients with that insurance either*.”

During member checking, the average level of participant agreement with the theme and its description was 5.8 and 5.3, respectively, on a 7-point Likert scale, falling between “somewhat agree” and “agree.” Participants recognized that these factors reduce NP reach in community, ambulatory urgent care, and acute care settings for vulnerable populations such as children, women, and older people. Collectively, the key constructs of this theme reflect the constraints on the potential impact of NPs on health equity, health disparities, and ultimately, population health outcomes.

### Theme 2: The distinct NP approach is an untapped opportunity to increase equity and reduce disparities in low-integration settings

This theme reflects participants’ recognition of the unique potential of the NP approach. They reflected on the potential benefits of NP care in settings where the role is underutilized. Participants highlighted that the NP approach to care is distinct from that of other healthcare professionals, and integration of NP roles can improve outcomes, advance health equity, and reduce disparities. In addition, they explicitly noted key elements of NP-patient relationships that support equity and reduce disparities.

The NP holistic, patient-centered model includes working with community resources to provide support in and out of the care setting. This is an essential strategy for reaching underserved populations. As **Participant 14** said, “*The NP approach is holistic and patient-centered. It emphasizes trust as foundational*,* applies therapeutic communication*,* addresses social and structural determinants of health*,* and collaborates with community resources to optimize support in the patient’s own environment and at home.”* Additionally, NPs use the therapeutic patient relationship as the cornerstone of health promotion and disease prevention, in contrast to other approaches that tend to prioritize medication or surgical treatment. As **Participant 1** noted: “*Nurse practitioners generally speak more with patients and explain better and will take the time to make sure that the patients understand what is being explained. This allows for greater understanding and compliance among patients from all walks of life*.”

Participants noted that NP practice emphasizes building relationships grounded in trust and therapeutic communication, and it considers social and structural determinants of health as foundational. **Participant 2** stated that “*It’s important to describe the role of trust here. NPs have high patient satisfaction ratings*,* and many patients tend to trust NPs over [other practitioners]*.” **Participant 9** said, “*NPs have a highly unique training and professional background that emphasizes caring for the “whole person”*,* not just their presenting disease process.*”

In contrast, other care models often focus solely on system- and illness-based care. Holistic and culturally sensitive care is standard in NP practice, and the collaborative development of care plans with patients is central to the approach, contributing to reduced disparities. **Participant 3** noted, *“[the NP approach] can include things like recognizing unmet social determinants of health and referring to appropriate community resources*,* assessing the patient’s home situation and social support available*,* and in many cases spending more time with patients and families rather than just a quick assessment.”* **Participant 13** said: “*In a school-based health clinic*,* the NP was fully integrated with their own patient panel and a team…that allowed the NP to address social determinants of health that impact disparities.*” Another said, *“For example in women’s health- the male HCP may not be the best address for women of Arab or Jewish culture. The women’s health NP can address this.”*

Participants noted that NPs are more likely to serve racial and ethnic minorities and geographically underserved communities, and that practice restrictions significantly limit their ability to do so. **Participant 4** said, “*NPs deliver care in underserved areas and racial and ethnic minorities. Allowing NPs to deliver care to the fullest extent of their training and integrating them into the system will have a major impact.*” However, in systems where NPs are not integrated to their full capacity, this unique approach is constrained, thus mitigating its potential to address health disparities and improve outcomes for populations who might benefit the most. **Participant 11** said, “*Health systems often do not prioritize the integration of NPs into practice. When NPs are not well integrated into practice*,* the application of the unique NP approach is limited.”*

During member checking, the average level of participant agreement with the theme and its description was 5.7 and 5.4, respectively, on a 7-point Likert scale, indicating strong agreement. The participants believed that NPs represent an untapped opportunity: where role integration is limited, there is great potential to improve access, equity, and outcomes in underserved settings. With enhanced integration, NPs could apply their holistic, patient-centered, and community-oriented model to achieve these goals.

## Discussion

In this qualitative, survey-based study, experts were asked to describe how NP integration affects the relationship between NP care and reduced health disparities for underserved populations. Two themes were identified: (1) Poor integration and limited role autonomy in the collaborating organization restrict NP capacity to promote equity and decrease disparities; and (2) The distinct NP approach is an untapped opportunity to increase equity and reduce disparities in low-integration settings.

Much of the US national-level discussion on policies affecting NP integration relates to state-level practice authorities. State-level authorities must permit sufficient independence to provide care to the fullest extent of an NP’s education and training [13]. Nevertheless, research shows that organizations often impose additional limitations on the NP scope of practice, restricting roles even further than required by state regulations [35]. These organizational policies are often enacted due to concerns that NPs may present a potential risk to patient care quality without oversight and practice restrictions, despite a lack of evidence to support this [36]. The participants in this study identified the trickle-down role of organizational policy and state-level scope of practice in NP integration. This notion is reflected in the first theme: *Poor integration and limited organizational role design restrict NP capacity to promote equity and decrease disparities.*

Research has shown that NPs effectively bridge between the health system and patient culture [37–39]. This unique niche relates to five NP attributes—holistic approach to care, developing partnerships with patients, personalism, professional standards, and training in cultural brokering [37]. Our study results suggest that the unique outcomes of NP care, as noted in the literature [40], may not be optimized without greater attention to policies that affect NP integration. This notion is reflected in the second theme: *The distinct NP approach is an untapped opportunity to increase equity and reduce disparities in low-integration settings.*

Claims that NPs contribute to reducing health disparities often point to their strong educational preparation and broad scope of practice. However, a closer look at the literature reveals that some of these claims are based on assumptions or opinion pieces rather than actual data [14]. Our study helps address that gap by drawing on the experience of experts in NP practice and integration. The participants consistently described how NP roles can directly impact equity - through increased access, stronger patient relationships, cultural sensitivity, and continuity of care. Our results offer a consensus among those positioned to observe the connection between NP practice and equity outcomes. In doing so, this study gives weight and credibility to arguments that, until now, have lacked firsthand evidence.

Beyond the local US contexts of our participants, our findings resonate with the broader Sustainable Development Goals, particularly the pursuit of universal health coverage. Many countries are seeking practical and sustainable ways to expand access to high-quality primary care, particularly in underserved or rural areas. The integration of NPs, as described by the experts in this study, not only fills workforce gaps but also improves access to affordable care that is grounded in holistic, patient-centered care and long-term patient relationships. For countries still developing or revising their health system structure, there is value in looking beyond the usual economic and structural frameworks and considering how professional roles, like that of the NP, can help shape more equitable systems and reduce disparities from the ground up.

### Recommendations for future research and policy

Participants provided feedback indicating that the ability of NPs to reduce health disparities relies on the effective integration into the healthcare system. These findings reinforce the hypothesis that integrating NPs is a key implementation issue that warrants further research. Empirical assessment of the impact of NP implementation on reducing inequities and health disparities may contribute to the lack of studies addressing this issue. As such, application of an implementation science lens may be beneficial when conceptualizing NP integration [41]. For example, using the Consolidated Framework for Implementation Research 2.0 [42] may allow researchers to identify the outer setting, inner setting, personal attributes, and implementation process that affect the integration of NPs. Furthermore, implementation science can inform research and policy development to improve the integration of NPs.

Additionally, investment in NP integration is crucial for developing equitable and efficient healthcare systems [43]. Without proper investment at the system level, NPs will remain underutilized. Strategic investment in regulatory alignment, role clarity, interprofessional education, branding, and organizational readiness will enhance the integration of NPs and strengthen the overall healthcare workforce, ultimately impacting health inequities and disparities. The NP Integration Model, used in this study, can help guide regulatory alignment and efficacious NP integration policy development [10, 11].

### Strengths and limitations

A key strength of our study is the use of member checking. Participants provided detailed feedback after analyzing their initial responses to the questions. Another strength is the implementation science lens, provided through the NP Integration Model. It is uncommon to apply implementation science to workforce implementation, or, in this case, integration. Finally, participants consisted of both scholars and practice experts, which enhanced the breadth and transferability of this study.

Regarding limitations, qualitative research is often richer when collected in interviews or focus groups. Survey-based responses to questions limit the investigators from asking probing and follow-up questions. Another limitation is the reliance on participant identification from the advisory panel. The panel may have nominated participants whom they know, resulting in a less diverse sample. Nevertheless, we collected demographics and reviewed advisory panel nominations to somewhat reduce the probability that the sample lacks sufficient diversity.

## Conclusions

Strengthening NP integration is essential for advancing the Sustainable Development Goals by expanding access to equitable, high-quality care. Despite the large body of evidence showing that NP care improves health outcomes, strategic and efficacious NP integration into care models may represent a missed opportunity to improve access, reduce inequities, and address health disparities. Investment and attention should be given to policies and practices that impact the integration of NPs, ensuring that the desired outcomes of introducing NPs into care models are achieved.

## Data Availability

The datasets used and/or analyzed during the current study are available from the corresponding author on reasonable request.

## Abbreviations

NP: Nurse practitioner

## References

[1] Kinchen E. Holistic nursing values in nurse practitioner education. Int J Nurs Educ Scholarsh. 2019;16(1). 10.1515/ijnes-2018-0082 31539360

[2] Poghosyan L, Liu J, Chen JL, Flandrick K, McMenamin A, Porat-Dahlerbruch J et al. Racial disparities in hospitalization and neighborhood deprivation among medicare beneficiaries. Health Affairs Scholar. 2025. 10.1093/haschl/qxaf010 PMC11803629 39927097

[3] Carron R. Health disparities in American Indians Alaska. The Nurse Practitioner. 2020;45(6):26–32. 32433371 10.1097/01.NPR.0000666188.79797.a7

[4] Crear-Perry J, Correa-de-Araujo R, Lewis Johnson T, McLemore MR, Neilson E, Wallace M. Social and structural determinants of health inequities in maternal health. J Womens Health (Larchmt). 2021;30(2):230–5. 33181043 10.1089/jwh.2020.8882 PMC8020519

[5] Winsley S, Link D. Breaking down barriers. J Nurse Practitioners. 2020;16(3):240–1.

[6] DesRoches CM, Clarke S, Perloff J, O’Reilly-Jacob M, Buerhaus P. The quality of primary care provided by nurse practitioners to vulnerable medicare beneficiaries. Nurs Outlook. 2017;65(6):679–88. 28803624 10.1016/j.outlook.2017.06.007

[7] Xue Y, Poghosyan L, Lin Q. Supply and geographic distribution of geriatric physicians and geriatric nurse practitioners. JAMA Netw Open. 2024;7(11):e2444659. 39535794 10.1001/jamanetworkopen.2024.44659 PMC11561691

[8] World Health Organization and International Council of Nurses. State of the World’s Nursing: Investing in Education, Jobs, Leadership and Service Delivery. 2025.

[9] Porat-Dahlerbruch J, Poghosyan L, Ellen M. Nurse practitioner integration: insights into the next generation of policy and research. Int J Health Policy Manage. 2023;12(1):1–4. 10.34172/ijhpm.2023.7411 PMC10461900 37579374

[10] Porat-Dahlerbruch J, Chen I, Cahir K, Blumenthal NP. Adaptation of the nurse practitioner integration model for older adult primary care models. Geriatr Nurs. 2025;65(5):1–8. 10.1016/j.gerinurse.2025.103453 PMC12947783 40639073

[11] Porat-Dahlerbruch J, Poghosyan L, Blumenthal N, Ratz S, Ellen ME. Nurse practitioner integration: conceptual development to enhance application in policy and research. J Am Association Nurse Practitioner. 2022;34(10):1106–15. 10.1097/JXX.0000000000000761 35900920

[12] Brooks Carthon JM, Brom H, Nikpour J, Todd B, Aiken L, Poghosyan L. Supportive practice environments are associated with higher quality ratings among nurse practitioners working in underserved areas. J Nurs Regul. 2022;13(1):5–12. 36249162 10.1016/s2155-8256(22)00028-x PMC9555817

[13] Schorn MN, Myers C, Barroso J, Hande K, Hudson T, Kim J, et al. Results of a National survey: ongoing barriers to APRN practice in the united States. Policy Politics Nurs Pract. 2022;23(2):118–29. 10.1177/15271544221076524 35119332

[14] Porat-Dahlerbruch J, Clark R, Dutchess B, Blumenthal NP, Ellen ME. Factors affecting integration of the nurse practitioner workforce into health systems: a Delphi consensus study. BMC Health Serv Res. 2025;25(1). 10.1186/s12913-025-12929-w PMC12128251 40457343

[15] Poghosyan L, Liu J, Perloff J, D’Aunno T, Cato KD, Friedberg MW, et al. Primary care nurse practitioner work environments and hospitalizations and ED use among chronically ill medicare beneficiaries. Med Care. 2022;60(7):496–503. 35679173 10.1097/MLR.0000000000001731 PMC9202077

[16] McMenamin A, Turi E, Schlak A, Poghosyan L. A systematic review of outcomes related to nurse practitioner-delivered primary care for multiple chronic conditions. J Med Care Res Rev. 2023:10775587231186720. 10.1177/10775587231186720 PMC10784406 37438917

[17] O’Reilly-Jacob M, Featherston KG, Barnes H, Xue Y, Poghosyan L. Socioeconomic characteristics of communities with primary care practices with nurse practitioners. JAMA Netw Open. 2025;8(2):e2462360. 40019758 10.1001/jamanetworkopen.2024.62360 PMC11871540

[18] Norful AA, de Jacq K, Liu J, Ye S, Khadka S, Poghosyan L. The impact of nurse practitioner and physician assistant workforce supply on Medicaid-related emergency department visits and hospitalizations. J Am Assoc Nurse Pract. 2021;33(12):1190–7. 33534285 10.1097/JXX.0000000000000542 PMC8363165

[19] Maier CB, Aiken LH, Busse R. Nurses in advanced roles in primary care: policy levers for implementation. Paris, France: OECD Publishing; 2017.

[20] Torrens C, Campbell P, Hoskins G, Strachan H, Wells M, Cunningham M, et al. Barriers and facilitators to the implementation of the advanced nurse practitioner role in primary care settings: A scoping review. Int J Nurs Stud. 2020;104. 10.1016/j.ijnurstu.2019.103443 32120089

[21] Poghosyan L, Courtwright S, Flandrick KR, Pollifrone MM, Schlak A, O’Reilly-Jacob M, et al. Advancement of research on nurse practitioners: setting a research agenda. Nurs Outlook. 2023;71(5). 10.1016/j.outlook.2023.102029 PMC10810357 37619489

[22] Belton I, MacDonald A, Wright G, Hamlin I. Improving the practical application of the Delphi method in group-based judgment: A six-step prescription for a well-founded and defensible process. Technol Forecast Soc Chang. 2019;147:72–82.

[23] Toma C, Picioreanu I. The Delphi technique: methodological considerations and the need for reporting guidelines in medical journals. Int J Public Health Res. 2016;4(6):47–59.

[24] Morse JM. Critical analysis of strategies for determining rigor in qualitative inquiry. Qual Health Res. 2015;25(9):1212–22. 26184336 10.1177/1049732315588501

[25] U.S. Department of Health and Human Services. Health equity and health disparities environmental scan. Rockville, MD: U.S. Department of Health and Human Services, Office of the Assistant Secretary for Health, Office of Disease Prevention and Health Promotion; 2022. December 14, 2020.

[26] Bradley EH, Curry LA, Devers KJ. Qualitative data analysis for health services research: developing taxonomy, themes, and theory. Health Serv Res. 2007;42(4):1758–72. 17286625 10.1111/j.1475-6773.2006.00684.x PMC1955280

[27] Elo S, Kyngas H. The qualitative content analysis process. J Adv Nurs. 2008;62(1):107–15. 18352969 10.1111/j.1365-2648.2007.04569.x

[28] Olmos-Vega FM, Stalmeijer RE, Varpio L, Kahlke R. A practical guide to reflexivity in qualitative research: AMEE guide 149. Med Teach. 2022:1–11. 10.1080/0142159X.2022.2057287 35389310

[29] O’Brien BC, Harris IB, Beckman TJ, Reed DA, Cook DA. Standards for reporting qualitative research: A synthesis of recommendations. Acad Med. 2014;89(9):1245–51. 24979285 10.1097/ACM.0000000000000388

[30] Campbell R, Goodman-Williams R, Feeney H, Fehler-Cabral G. Assessing triangulation across methodologies, methods, and stakeholder groups: the joys, woes, and politics of interpreting convergent and divergent data. Am J Evaluation. 2018;41(1):125–44.

[31] Walter N, Cohen J, Holbert RL, Morag Y. Fact-Checking: A Meta-Analysis of what works and for whom. Political Communication. 2019;37(3):350–75.

[32] Guba EG. Annual review paper: criteria for assessing the trustorthiness of naturalistic inquiries. Educational Communication Technol. 1981;29(2):75–91.

[33] Ahmed SK. The pillars of trustworthiness in qualitative research. J Med Surg Public Health. 2024;2.

[34] Miles MB, Huberman AM, Saldaña J. Qualitative data analysis: A methods sourcebook. 3rd ed. Los Angeles (CA): SAGE; 2014.

[35] Poghosyan L, Aiken LH. Maximizing nurse practitioners’ contributions to primary care through organizational changes. J Ambul Care Manage. 2015;38(2):109–17. 25748259 10.1097/JAC.0000000000000054

[36] National Academies of Sciences, Engineering, and Medicine. The future of nursing 2020–2030. National Academy of Medicine; National Academies of Sciences, Engineering, and Medicine; 2021. 34524769

[37] Matteliano MA, Street D. Nurse practitioners’ contributions to cultural competence in primary care settings. J Am Acad Nurse Practitioners. 2012;24(7):425–35. 10.1111/j.1745-7599.2012.00701.x 22735066

[38] Taylor K. Bridging the gap to health care access: the role of the nurse practitioner. Int Archives Public Health Community Med. 2023;7(1):1–3.

[39] Villarruel AM, Sochalski J. Advancing primary care with underserved communities: a case study of the Leonard A. Lauder community care nurse practitioner program. Natl Acad Med Perspect. 2023:1–3. 10.31478/202302a PMC10238100 37273460

[40] Kilpatrick K, Savard I, Audet L-A, Costanzo G, Khan M, Atallah R, et al. A global perspective of advanced practice nursing research: A review of systematic reviews. PLoS ONE. 2024;19(7):e0305008. 38954675 10.1371/journal.pone.0305008 PMC11218965

[41] Porat-Dahlerbruch J, Ratz S, Aaron E, Ellen M. Understanding factors affecting the integration of geriatric nurse practitioners into health systems. J Am Association Nurse Practitioners. 2023;35(12):813–25. 10.1097/JXX.0000000000000937 37610786

[42] Damschroder LJ, Reardon CM, Widerquist MAO, Lowery J. The updated consolidated framework for implementation research based on user feedback. Implement Sci. 2022;17(1):75. 36309746 10.1186/s13012-022-01245-0 PMC9617234

[43] Porat-Dahlerbruch J, Boyd J, Fighel H. Investing in the advanced practice nursing workforce to improve health system responses to armed conflict. Int Nurs Rev. 2025;72(3):e70074. 40682315 10.1111/inr.70074 PMC12274788

